# Distinct Effects of Lexical and Semantic Competition during Picture Naming in Younger Adults, Older Adults, and People with Aphasia

**DOI:** 10.3389/fpsyg.2016.00813

**Published:** 2016-06-02

**Authors:** Allison E. Britt, Casey Ferrara, Daniel Mirman

**Affiliations:** ^1^Department of Psychology, Drexel UniversityPhiladelphia, PA, USA; ^2^Moss Rehabilitation Research InstituteElkins Park, PA, USA

**Keywords:** word selection, lexical access, semantics, picture naming, aging, aphasia, left prefrontal cortex

## Abstract

Producing a word requires selecting among a set of similar alternatives. When many semantically related items become activated, the difficulty of the selection process is increased. Experiment 1 tested naming of items with either multiple synonymous labels (“Alternate Names,” e.g., gift/present) or closely semantically related but non-equivalent responses (“Near Semantic Neighbors,” e.g., jam/jelly). Picture naming was fastest and most accurate for pictures with only one label (“High Name Agreement”), slower and less accurate in the Alternate Names condition, and slowest and least accurate in the Near Semantic Neighbors condition. These results suggest that selection mechanisms in picture naming operate at two distinct levels of processing: selecting between similar but non-equivalent names requires two selection processes (semantic and lexical), whereas selecting among equivalent names only requires one selection at the lexical level. Experiment 2 examined how these selection mechanisms are affected by normal aging and found that older adults had significantly more difficulty in the Near Semantic Neighbors condition, but not in the Alternate Names condition. This suggests that aging affects semantic processing and selection more strongly than it affects lexical selection. Experiment 3 examined the role of the left inferior frontal gyrus (LIFG) in these selection processes by testing individuals with aphasia secondary to stroke lesions that either affected the LIFG or spared it. Surprisingly, there was no interaction between condition and lesion group: the presence of LIFG damage was not associated with substantively worse naming performance for pictures with multiple acceptable labels. These results are not consistent with a simple view of LIFG as the locus of lexical selection and suggest a more nuanced view of the neural basis of lexical and semantic selection.

## Introduction

Producing a word requires selecting a single candidate word from a set of similar and related alternatives (e.g., Levelt et al., 1999; Howard et al., 2006; Dell et al., 2013; Walker and Hickok, 2015). These alternatives may include words that describe the concept at different levels in a taxonomic hierarchy (e.g., beagle–dog–mammal–animal), synonyms (e.g., sofa–couch), and words that refer to similar objects or concepts (e.g., eagle–falcon; sometimes called “semantic neighbors,” e.g., Mirman, 2011). Numerous studies have shown that activation of semantically related alternatives makes selection more difficult as reflected by slower response times and lower accuracy (e.g., Schnur et al., 2006; Bormann, 2011; Mirman, 2011; Roelofs et al., 2012; Shao et al., 2015). This selection process appears to engage ventro-lateral prefrontal cortex (VLPFC, also sometimes described as inferior frontal gyrus, IFG), typically left-lateralized for word-based tasks. That is, when selection is more difficult, neurologically intact individuals show greater activation of VLPFC/left inferior frontal gyrus (LIFG) and individuals with damage in this area are particularly prone to errors (e.g., Thompson-Schill et al., 1997, 1998; Schnur et al., 2009; Snyder et al., 2011, 2014; Mirman and Graziano, 2013).

The present experiments build on this prior research and investigate whether there is separate selection at lexical and semantic levels or a single lexical-semantic selection step. In prior research, the alternatives for selection were often both semantically and lexically different, so it was not possible to distinguish them. Here, we distinguish two selection scenarios: (1) selection among semantically distinct alternatives (i.e., semantic neighbors), which differ both semantically and lexically, and (2) selection of semantically equivalent lexical alternatives (i.e., synonyms). To do this, we used an “underdetermined object naming” task – naming of pictures that have multiple correct or acceptable names.

Name agreement is typically defined as the percentage of individuals who produce the same name for a given picture. Many studies have demonstrated a strong effect of name agreement on naming latency (e.g., Snodgrass and Yuditsky, 1996; Barry et al., 1997; Kremin et al., 2000; Bonin et al., 2002; Bates et al., 2003; Alario et al., 2004; Weekes et al., 2007) and fluency (Schnadt and Corley, 2006; Hartsuiker and Notebaert, 2010). Naming low name agreement pictures is also associated with increased left IFG activation (Kan and Thompson-Schill, 2004) and larger N2 ERP amplitude in anterior regions (Shao et al., 2014). Novick et al. (2009) reported that an individual with restricted damage to LIFG had particular difficulty with naming low agreement pictures (their Experiment 2) as well as difficulty in other selection-demanding tasks. Together, these studies suggest that naming low name agreement pictures induces greater selection demands and greater recruitment of neural systems involved in selection or competition resolution.

The behavioral effects of name agreement have been well-documented, but the reasons for a picture’s low name agreement rating are often not addressed. Vitkovitch and Tyrrell (1995) compared naming latencies for objects with three types of name disagreement: abbreviations (e.g., “phone”–“telephone”), multiple names (e.g., “couch”–“sofa”), and incorrect names (e.g., “spider”–“ant”). They found a name agreement effect for the multiple and incorrect names conditions, but not the abbreviations condition, suggesting that lexical-semantic selection is more difficult than selecting between abbreviated/elaborated names for the same concept. An ERP study found that P1 amplitude was associated with uncertainty about picture identity and N2 amplitude was associated with selection between multiple names (Cheng et al., 2010), suggesting an early visual object recognition process (indexed by P1) and a later lemma selection process (indexed by N2).

In sum, prior studies provide reasons to believe that selection takes place at the lexical level, the semantic level, or possibly both. The current study compared naming of high name agreement pictures against two kinds of low name agreement pictures. The first was “Alternate Names” pictures, which have multiple correct, synonymous labels. Norming was used to ensure that the alternate names were considered equivalent. The second was “Near Semantic Neighbors” pictures, for which individuals produced responses that were closely semantically related but not equivalent (these are a refined version of “incorrect” or “picture uncertainty” categories from previous studies). Although there is technically only one correct description of pictures in this condition, two or more very similar concepts may be virtually indistinguishable for many individuals.

Critically, any pictures for which individuals provided clearly incorrect, unrelated responses were excluded to eliminate visual ambiguity as a source of low name agreement (see Preliminary Study for details of stimulus norming and selection). Thus, the first goal of the current study was to replicate previous name agreement effects using stimuli that excluded any confusing or unclear pictures in order to localize the effects to lexical-semantic selection.

The second goal was to examine whether the selection takes place at a single lexical-semantic level or distinct lexical and semantic levels. Two plausible hypotheses predict opposite results. The first is based on semantic similarity. Items in the Alternate Names conditions have multiple responses that are equivalent in meaning and therefore more similar than responses for items in the Near Semantic Neighbors condition. That is, selecting between “sofa” and “couch” should be more difficult than selecting between “turtle” and “tortoise” because the former pair are more similar than the latter, just as the “turtle”–“tortoise” selection should be more difficult than selecting “trampoline,” which does not have any very near semantic neighbors. In contrast, a two-level (semantic and lexical) selection process makes the opposite prediction: alternate names selection (“sofa”–“couch”) involves only lexical selection because the semantic representations are identical, so it should be easier than near semantic neighbors selection (“turtle”–“tortoise”), which requires both semantic and lexical selection (different semantic representations and different lexical items). The first two goals were addressed in Experiment 1.

Experiment 2 replicated and extended the findings from Experiment 1 by examining how lexical and semantic selections are affected in typical healthy aging. Some prior studies have reported larger effects of selection for older adults compared to younger adults. For example, older adults exhibit larger effects of phonological neighborhood density in spoken word recognition (Sommers, 1996; Sommers and Danielson, 1999; Taler et al., 2010; Botezatu et al., 2015). LaGrone and Spieler (2006) found that older adults were more sensitive to name agreement, though (like other studies discussed above) they did not distinguish between visual, lexical, and semantic sources of name disagreement. In contrast, compared to younger adults, older adults do not seem to be more sensitive to interference from recently named pictures (Belke and Meyer, 2007; Gordon and Cheimariou, 2013; Mulatti et al., 2014). Thus, it is not clear whether older adults have greater difficulty with selection or, if they do, at what level this difference emerges. In Experiment 2, lexical and semantic selection was examined in older adults and compared to the younger adult data from Experiment 1.

The selection processes examined in Experiments 1 and 2 are thought to engage the LIFG (or VLPFC). Several studies have found that damage to the LIFG impairs selection in tasks such as blocked cyclic naming (Schnur et al., 2006, 2009) and generating an appropriate verb in response to a noun prompt (Thompson-Schill et al., 1998). Each of these tasks compares performance in a control condition with lower selection demands to a condition with higher selection demands. In blocked cyclic naming, that comparison is between naming a semantically mixed or heterogeneous block of pictures vs. naming a block of semantically related pictures; in verb generation, that comparison is between a noun prompt that is strongly associated with only one verb vs. a noun prompt that is associated with multiple verbs. The critical findings were that LIFG damage had a greater effect on performance in the condition with the greater selection demands. These results align well with functional neuroimaging data showing increased LIFG/VLPFC activation in neurologically intact participants under greater selection demands in these same tasks (Thompson-Schill et al., 1997; Schnur et al., 2009; Snyder et al., 2011, 2014) as well as other tasks, including naming pictures with low name agreement (Kan and Thompson-Schill, 2004; Novick et al., 2009). On the basis of these prior results, we predicted that participants with LIFG lesions should also have particular difficulty naming our low name agreement pictures. Prior studies focused on semantically driven selection demands (naming a block of semantically related pictures, selecting from verbs associated with a common noun, etc.) so this prediction was strongest for the Near Semantic Neighbors condition (relative to the High Name Agreement condition). However, the theoretical interpretation of these effects has been tied to a central selection system that is independent of specific processing levels (implicit in most of the work cited above and more explicitly discussed in Snyder et al., 2007; Novick et al., 2010; Munakata et al., 2011), so an analogous effect for the Alternate Names condition was also predicted.

Together, these three experiments provided a broad investigation of lexical and semantic selection processes during picture naming: dissociating the two levels of selection (Experiment 1), and examining how they are affected in normal aging (Experiment 2) and by damage to left frontal brain regions (Experiment 3).

## Preliminary Study: Stimulus Norming and Selection

An initial set of candidate stimuli was selected from a set of 384 pictures that had undergone name agreement norming for a previous study. Picture naming data had been collected for these pictures from 20 individuals via Amazon Mechanical Turk (16 female, average age = 34). These picture naming responses were scored for name agreement (proportion of participants who provided the most common name) and “concept agreement”: the proportion of participants who indicated correct general identification of the concept of a picture, even if the name selected was not the most common among the group. For example, labeling a picture of a piece of chalk as “straw” or a picture of a bracelet as “candies” indicates visual rather than semantic or lexical ambiguity. In contrast, a response of “coat” for an item most respondents called “jacket” indicates agreement about the general concept represented by the picture but disagreement about the name.

To be considered for inclusion in the present study, low name agreement pictures had to have name agreement ≤75% (i.e., the most common name was provided by at most 75% of participants) and concept agreement ≥80% (i.e., at least 80% of participants correctly identified the pictured concept). By these definitions, 16.4% of the 384 pictures had low name agreement. Only about 2% of pictures had low concept agreement; however, of the pictures with low name agreement, 11.1% had low concept agreement. That is, lack of concept agreement contributes to low name agreement, suggesting that prior studies of name agreement effects may have mixed lexical-semantic selection and visual ambiguity effects (unless they controlled for this without reporting it).

Fifty-six pictures met the name and concept agreement criteria (14.6% of all pictures), and forty were selected as potential stimuli based on the nature of the responses from the name agreement norming study. Items for which name disagreement was strongly related to dialectal differences (e.g., “soda”–“pop”) were not included. Items were tentatively assigned to one of two conditions: pictures that received synonymous labels were assigned to the Alternate Names condition; pictures that received non-equivalent but closely related labels were assigned to the Near Semantic Neighbors condition.

Twenty additional participants were recruited from Mechanical Turk (13 female, average age = 43) for two further norming tasks. The first was a synonymy judgment task in which participants were presented with two words and asked to indicate by Yes/No response whether the two words “mean the same thing.” For each picture item, all responses provided by more than one participant in the name agreement norming study were included in every combination of pairs. For example, participants were asked whether the pairs “branch” and “stick,” “branch” and “twig,” and “stick” and “twig” mean the same thing. No pictures were presented during this task and word pairs were presented in a random order.

In the second task, participants were shown a picture with four words and asked to select all words that were a good description of the picture. Any response provided by more than one participant in the name agreement norming was included. If there were fewer than four responses, related but incorrect items were included. For example, participants were asked to judge whether the words “turtle,” “tortoise,” “frog,” and “lizard” were good descriptions for a picture that all previous participants had labeled “turtle” or “tortoise.”

For the Alternate Names condition, items should have alternate labels judged as equivalent and all endorsed as good descriptions of a picture. For example, 95% of participants indicated that both “coins” and “change” were good descriptions of one of the pictures, and 85% reported that “coins” and “change” mean the same thing. Thus, the two words seem to be alternate labels used to refer to the same concept. Responses for items in the Near Semantic Neighbors condition should also all be endorsed as good descriptions of the picture but labels should not be judged to mean the same thing. For example, the majority of participants (75%) indicated that both “alligator” and “crocodile” were good descriptions of a depicted animal, but only 15% reported that they thought “alligator” and “crocodile” meant the same thing. This suggests that the two labels refer to two different but very similar concepts. Based on this second set of norming results, three items were excluded from each condition.

The final set of stimuli included 17 items in each of the two low name agreement conditions and 34 additional high name agreement items, which had 100% name agreement in the original name agreement norming study. Items in the Alternate Names and Near Semantic Neighbors conditions differed significantly on synonymy ratings. They also differed on average number of names selected as a good description during norming, though this slight difference disappeared in our lenient accuracy coding of the naming responses (see below). Critically, for both groups of low name agreement pictures, participants consistently indicated that more than one name was a good description of the picture, indicating an increased need for name selection. The three conditions were also matched on word length (number of phonemes), word frequency (SUBTLEX frequency per million words; Brysbaert and New, 2009), and objective visual complexity (.gif image file size in kilobytes; Székely and Bates, 2000). For low name agreement items, weighted length and frequency measurements were calculated based on the proportion of participants who produced each acceptable response^1^. For high name agreement items, length and frequency were determined based on the single response provided by all participants. The Alternate Names and Near Semantic Neighbors conditions were equated on h-index, a name agreement measure that takes into account the number of participants who provide each of the different responses for an item (greater naming agreement among subjects results in an h-index closer to 0; for a more detailed description, see Snodgrass and Vanderwart, 1980). Characteristics of these critical stimuli are provided in **Table 1** and all low name agreement pictures and accepted labels are listed in the Supplementary Material.

**Table 1:** Mean characteristics of critical stimuli (*SD* in parentheses).

	High Name Agreement	Alternate Names	Near Semantic Neighbors
Number of pictures	34	17	17
Name agreement	100% (0%)	65.6% (9.3%)	57.1% (15.6%)
h-index	–	1.01 (0.28)	1.03 (0.44)
Synonymy	–	0.71 (0.23)	0.18 (0.14)
Number of labels selected during norming	–	2.16 (0.35)	1.80 (0.50)
Number of labels accepted as correct for scoring purposes	1.0 (0)	2.41 (0.62)	2.47 (0.80)
Length	5.21 (1.37)	5.51 (1.48)	5.45 (1.25)
Word frequency	15.18 (19.33)	20.20 (28.38)	12.66 (15.86)
Objective visual complexity	6.37 (1.62)	6.89 (2.35)	6.73 (2.51)

## Experiment 1

### Methods

#### Participants

Fifty Drexel University students (36 female, 14 male) participated in the study for course credit or payment. Average age of participants was 20 (range: 18–25). All had normal or corrected-to-normal hearing and vision and reported no history of neurological, cognitive, or language deficits. Thirty-four participants were native English speakers. Twelve were native bilinguals exposed to English and another language from birth. The remaining four were non-native English speakers but learned English by age 5 at the latest. All participants identified English as their current primary language.

#### Procedure

Participants saw pictures appear on a computer screen one at a time and were instructed to name the item using one word. Each item was preceded by a 380-ms tone to indicate the start of the trial. The study began with five practice trials, followed by the 68 experimental trials presented in a random order. No time limit was imposed, and participants were allowed to skip any item for which they could not think of a name.

#### Scoring

Naming latencies were hand-coded oﬄine by the experimenter using Praat. Latencies included the time from the start of the tone to the onset of the participant’s response, not including any filler words or articles prior to the response word.

Lenient accuracy scoring was used to account for the multiple acceptable responses for low name agreement items. Any alternate name or near semantic neighbor (depending on the condition) provided by more than one participant in the name agreement norming study was coded as correct in the current study. Novel responses (those provided by experiment participants but not in the norming study) were considered accurate based on consensus judgment of the experimenters (3.3% of trials). If participants provided more than one response for a given trial, only the first response was coded.

Responses judged to be errors were coded as one of seven categories, described in **Table 2**. Of particular importance is the differential coding of semantically related responses for the two low name agreement conditions. Any semantically related response (including “mixed” responses that were both semantically and phonologically related) provided by more than one participant in the norming study was coded as correct for items in the Near Semantic Neighbors condition. However, near semantic neighbors were not coded as correct for the Alternate Names condition, since they cannot be considered equivalent, synonymous responses. Superordinate responses (e.g., “instrument” for “tuba”) were coded as errors in all conditions.

**Table 2:** Descriptions and examples of response categories used for accuracy coding.

Error category	Description	Example
Correct	Appropriate label for picture provided by at least two individuals in previous norming study; or any additional response judged to be accurate based on consensus judgment of the experimenters	For one picture: “jam” and “jelly” both considered correct (responses from norming study); also accepted additional response “preserves”
Phonological – Non-word	A non-word response that shares the initial phoneme and/or at least 50% of phonemes with the target word	“Thack” for “tack”
Phonological – Formal	A semantically unrelated real-word response that shares the initial phoneme and/or at least 50% of phonemes with the target word	“Tram” for “pram (carriage)”
Semantic	A semantically related response. All counted as errors in Alternate Names condition; only more distantly related responses not occurring in the norming name agreement study counted as errors in the Near Semantic Neighbors Condition	Alternate Names condition: “turkey” for “chicken” Near Semantic Neighbors condition: “jar” for “beaker”
Mixed	A semantically related response that shares the initial phoneme and/or at least 50% of phonemes with the target word (same condition differences described for semantic errors apply)	Alternate Names condition: “can opener” for “corkscrew” Near Semantic Neighbors condition: “sponge” for “squeegee”
Superordinate	Overarching category name of the target word	“Instrument” for “tuba”
Other	Semantically and phonologically unrelated responses or descriptions of the target word	“Marble” for “olives;” “something from under the sea” for “jellyfish”
No response	An indication of uncertainty about identity of picture with no response provided	“I don’t know what that is”

#### Data Analysis

Data were analyzed using multilevel regression with fixed effects of condition (High Name Agreement vs. Alternate Names vs. Near Semantic Neighbors) and crossed random effects of subjects and items (e.g., Baayen et al., 2008). Maximal random effect structures (Barr et al., 2013) were used whenever possible (random slopes of condition by subject and random intercepts by picture) and simplified if required for model convergence. Models were implemented in R version 3.2.3 (R Core Team, 2015) using lme4 package version 1.1.11 (Bates et al., 2015) and pairwise comparisons were evaluated using multcomp package version 1.4.4 (Hothorn et al., 2008). For analyses of naming latencies, outliers were identified by fitting an initial model to all qualifying trials, identifying trials with residuals more than 3 SD away from the mean and re-fitting the model with those trials excluded. This approach takes advantage of the multilevel regression model of group-level as well as participant-level and item-level differences to identify outliers that exceed even systematic individual participant and item variability. *P*-values were estimated using the normal approximation.

### Results

#### Naming Latency

**FIGURE 1:**
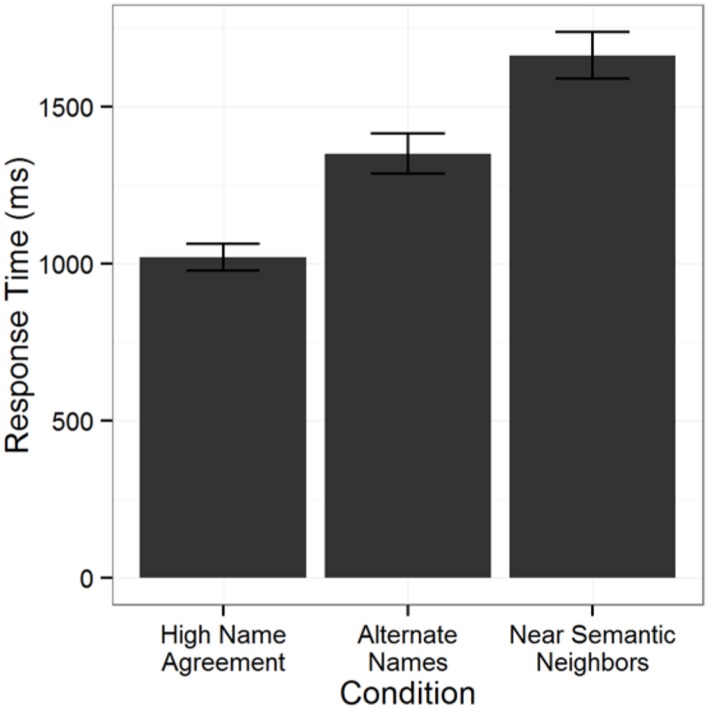
**Mean naming latencies in each condition (error bars indicate ±SE), with error trials and outliers excluded**.

Only correct response trials were considered for the initial latency analysis and one participant was excluded due to low overall accuracy (less than 80% correct). The initial model was fit to 3088 observations, then 1.6% of those were excluded as outliers and the model was refit to the remaining 3038 observations. Not surprisingly, naming latency was fastest in the High Name Agreement condition and slower in each of the two low name agreement conditions (**Figure 1**). Critically, naming latency was faster in the Alternate Names condition compared to the Near Semantic Neighbors condition (see **Table 3**, left column).

**Table 3:** Comparisons of condition differences in naming latency.

Condition comparison	Correct response trials	All response trials	Refined subset
High Name Agreement vs. Alternate Names	330 (74)^∗∗∗^	366 (89)^∗∗∗^	309 (56)^∗∗∗^
High Name Agreement vs. Near Semantic Neighbors	642 (82)^∗∗∗^	720 (96)^∗∗∗^	581 (70)^∗∗∗^
Alternate Names vs. Near Semantic Neighbors	313 (88)^∗∗^	354 (104)^∗∗^	272 (77)^∗∗^

*Values in cells indicate mean estimated differences (in ms) between conditions (SE in parentheses). ^∗∗^p < 0.001; ^∗∗∗^p < 0.0001.*

One possible concern is that the accuracy coding was somewhat subjective, with some semantically related responses being considered correct and others considered incorrect. To address this, we also analyzed latencies from all participants and all trials except those for which the participant gave no response (i.e., the most lenient possible accuracy coding: any response is considered correct). The initial model was fit to 3352 observations, 1.5% of which were excluded as outliers and the model was refit to the remaining 3303 observations. The same overall pattern of results was observed (**Table 3**, middle column), with latency significantly increasing from the High Name Agreement to the Alternate Names to the Near Semantic Neighbors condition.

The (relatively large) set of picture naming responses collected in this Experiment provides an additional source of name and concept agreement data. Eight low name agreement items had within-Experiment name agreement higher than 75% (“boat,” “tissues,” “coins,” “pasta,” and “cereal” from the Alternate Names condition; “jellyfish,” “turtle,” and “mango” from the Near Semantic Neighbors condition). Because closely related responses were all considered correct, accuracy is roughly equivalent to concept agreement and six low name agreement items had accuracy scores below 80% (“hourglass” and “corkscrew” from the Alternate Names condition; “chisel,” “tower,” “squeegee,” and “beaker” from the Near Semantic Neighbors condition), suggesting possibly low concept agreement. The data were re-analyzed with these items removed, leaving 10 low name agreement items in each of the two low name agreement conditions. In this subset of items, the conditions were still matched on word frequency, length, and h-index. Error trials and outliers (1.5% of 2564 observations) were excluded, and the same pattern of results emerged: fastest responses in the High Name Agreement condition, slower in the Alternate Names condition and slowest in the Near Semantic Neighbors condition (see **Table 3**, right column).

#### Accuracy

Lenient accuracy coding was used for both low name agreement conditions, allowing all viable responses to be considered correct (see above for details). For the high name agreement condition, only the single response produced during the norming study was considered correct. Analysis of accuracy across all 50 participants and trials revealed a pattern that was similar to that of latency. Accuracy was highest in the High Name Agreement condition (*M* = 99.0%; 95% CI = 97.9–99.5%), lower in the Alternate Names condition (*M* = 97.6%; 95% CI = 94.0–99.1%), and lowest in the Near Semantic Neighbor condition (*M* = 91.6%; 95% CI = 82.1–96.3%). A multilevel logistic regression model revealed that the Near Semantic Neighbors condition had statistically significantly lower accuracy than the other two conditions (compared to Alternate Names condition: *Estimate* = -1.33, *SE* = 0.626, *p* = 0.0334; compared to the High Name Agreement condition: *Estimate* = -2.22, *SE* = 0.553, *p* < 0.0001), but the difference between Alternate Names and High Name Agreement conditions was not statistically significant (*Estimate* = -0.885, *SE* = 0.575, *p* = 0.124).

#### Error Types

**FIGURE 2:**
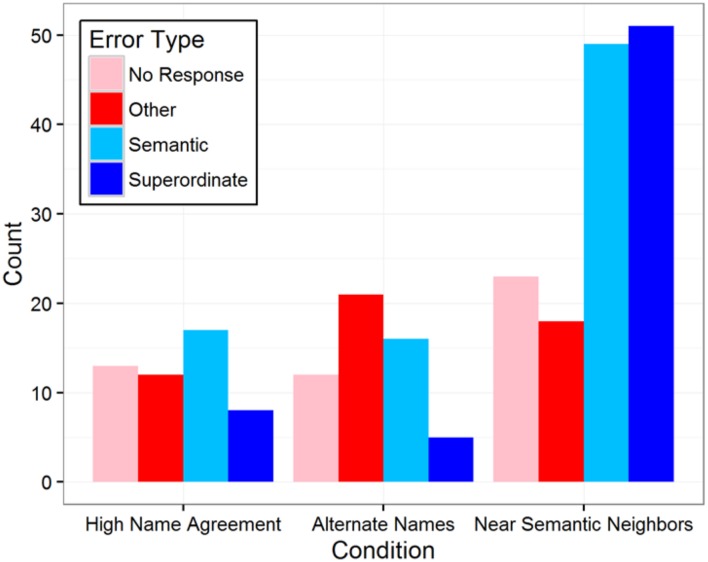
**Total number of errors as a function of error type and condition (excluding phonological and mixed errors)**.

In addition to condition differences in overall accuracy, there was also a different distribution of error types between the two low name agreement conditions (**Figure 2**). Phonological and mixed errors were very rare in all conditions (less than 10 occurrences of each). “Other” error types were similar across the conditions and there were slightly more “no response” errors in the Near Semantic Neighbors condition than the Alternate Names condition. The most striking difference in error type distributions was the much higher rates of semantic and superordinate errors in the Near Semantic Neighbors condition. Our lenient accuracy scoring strategy for the Near Semantic Neighbors condition means that these semantic errors were responses that were substantially different from the target, since any closely related responses would have been coded as correct. A chi-square goodness-of-fit test confirmed that the error type distributions differed between the conditions (across all three conditions: χ^2^(6, *N* = 245) = 28.7, *p* < 0.0001; between just the two low name agreement conditions: χ^2^(3, *N* = 195) = 24.2, *p* < 0.0001).

### Discussion

Pictures with low name agreement were named more slowly and less accurately than pictures with high name agreement, replicating previous effects of name agreement using a carefully selected set of stimuli in which name agreement was low but concept agreement was high. The high concept agreement makes it possible to rule out ambiguous or unclear pictures as a source of name disagreement, thus localizing the effect to lexical-semantic levels of processing.

Picture naming was substantially faster and more accurate for pictures with multiple equivalent names (“Alternate Names” condition, e.g., “pillar”–“column”) than for pictures with multiple non-equivalent, but closely related names (“Near Semantic Neighbors” condition, e.g., “jam”–“jelly”). These two conditions did not differ in h-index, a measure of name agreement. A single lexical-semantic selection step predicts that selecting between equivalent names should be most difficult because their representations are more similar than the representations of non-equivalent, but closely related names. This view is inconsistent with the present data. In contrast, separate semantic and lexical selection steps predict that selecting between equivalent names should be easier than selecting between non-equivalent names because the latter requires two selection processes (semantic and lexical) whereas the former only requires one selection (lexical, because the semantic representation is equivalent). Note that the dissociation of lexical and semantic selection does not require a modular or serial architecture, only that selection mechanisms operate at two distinct levels of processing, which may occur in parallel and involve bidirectional interactive activation.

In addition to overall speed and accuracy, the dissociation of lexical and semantic selection was supported by the observed differences in error type distributions between the two low name agreement conditions. Participants made strikingly more semantic and superordinate errors in the Near Semantic Neighbors condition than in the Alternate Names condition. For example, superordinate errors were fairly common for Near Semantic Neighbors items such as *chisel* (“tool”), *pastry* (“food,” “sweets,” “dessert”), *tower* (“building”), and *tuba* (“instrument”), but virtually non-existent Alternate Names items. This error pattern is consistent with our interpretation that the Near Semantic Neighbors condition induced substantially more selection difficulty specifically at the semantic level of processing – high proportions of superordinate errors suggest access to some accurate conceptual information but difficulty selecting among closely related semantic neighbors during naming of these types of items. Fewer semantically related errors in the Alternate Names condition suggests that the selection difficulty was more limited to lexical processing and that semantic selection was not needed to distinguish equivalent alternate labels.

## Experiment 2

This experiment extended the findings from Experiment 1 to examine how lexical and semantic selections are affected in typical healthy aging. As discussed in the Introduction, prior studies have reported mixed results regarding whether older adults have greater difficulty with selection or, if they do, at what level this difference emerges. Experiment 1 showed that our methods can distinguish between purely lexical selection and combined lexical and semantic selection, so Experiment 2 was designed to assess whether and at which level older adults have greater selection difficulty.

### Methods

The experimental materials, procedure, and response coding were the same as in Experiment 1. Multilevel regression was used to analyze the data, following the same approach as in Experiment 1, but including age group as a fixed effect and as a random slope term in the by-item random effects.

#### Participants

Twenty neurologically intact older adults were recruited to participate from the Moss Rehabilitation Research Registry and the Drexel University community. One was unable to participate due to scheduling constraints, so 19 older adults (16 female, 3 male) completed the study for payment. All were native English speakers with normal or corrected-to-normal hearing and vision and reported no history of neurological, cognitive, or language deficits. Two additional participants were excluded from analyses due to low score on the Mini-Mental State Exam (MMSE; Folstein et al., 1975), suggesting possible mild cognitive impairment. The remaining 17 participants had MMSE scores ≥26 (*M* = 28.4, range = 26–30). Average age of participants was 65 (range: 50–81)^2^.

### Results

**FIGURE 3:**
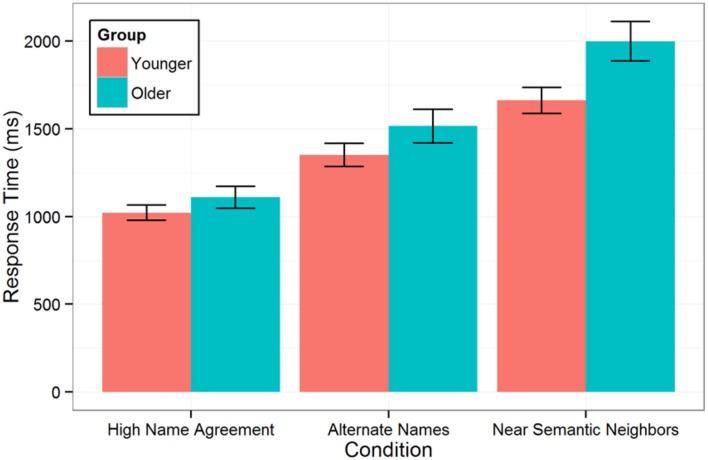
**Mean picture naming latency by condition and age group**. Error bars indicate ±SE.

Analyses of naming latencies followed the same approach as in Experiment 1. An initial model was fit to all correct response trials from the complete set of 65 participants (49 younger adults and 16 older adults). Of the 4079 observations in the initial model, 1.6% were identified as outliers on the basis of residual error greater than 3 SD and excluded, leaving 4013 observations. Older adults were slower to name pictures in all three conditions [model comparison for fixed effect of age group: χ^2^(1) = 4.78, *p* = 0.029], and this difference increased with increasing selection difficulty (**Figure 3**). The group-by-condition interaction indicated that the age group difference was statistically significantly larger in the Near Semantic Neighbors condition than the High Name Agreement condition (*Estimate* = 265, *SE* = 110, *p* = 0.023). The age group difference for the Alternate Names condition (175 ms) was intermediate – larger than in the High Name Agreement condition (92 ms) but smaller than in the Near Semantic Neighbors condition (357 ms) – not statistically significantly different from the High Name Agreement condition (*p* > 0.3) and marginally smaller than the Near Semantic Neighbors condition (*Estimate* = 182, *SE* = 108, *p* = 0.09).

**FIGURE 4:**
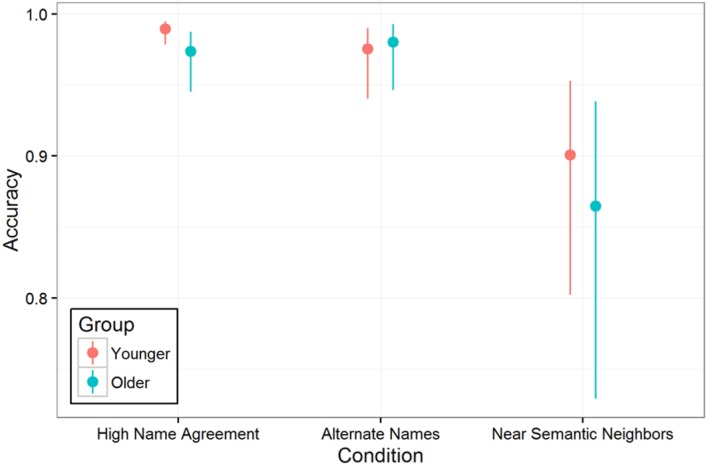
**Picture naming accuracy by condition and age group.** Vertical lines indicate 95% confidence intervals.

As in Experiment 1, naming accuracy was analyzed using multilevel logistic regression and including all trials from 67 participants (excluding two older adult participants with low MMSE scores, but including all other participants regardless of overall accuracy). The random effects were limited to random intercepts for participants and items to facilitate model convergence. Overall, older adults were not significantly less accurate than younger adults were [97.6% correct vs. 96.3% correct; model comparison for fixed effect of age group: χ^2^(1) = 2.1, *p* > 0.1], but there was a statistically significant interaction between age group and condition [model comparison for age group by condition interaction: χ^2^(2) = 9.65, *p* < 0.01]. This interaction appeared to be driven by a larger effect of age group on accuracy in the High Name Agreement condition than in the other two conditions^3^; indeed, the older adults were slightly more accurate at naming pictures in the Alternate Names condition than the younger adults were (**Figure 4**).

### Discussion

This experiment used naming of low and high name agreement pictures to examine the effect of typical aging on lexical and semantic selection. In Experiment 1, we found that young adults exhibit both lexical selection and semantic selection effects on response time: naming times were fastest for high name agreement pictures, slower when there were multiple semantically equivalent lexical alternatives (requiring lexical, but not semantic selection), and slowest when there were near semantic neighbors so that target word selection required both semantic and lexical selection. In Experiment 2, we collected additional data from older adults and found that older adults were slower to name pictures overall and this age-related difference monotonically increased with increasing selection demands: it was smallest in the High Name Agreement condition, larger in Alternative Names condition, and largest in Near Semantic Neighbors condition. This suggests that, in the context of picture naming, compared to younger adults, older adults have progressively more difficulty as lexical and semantic selection demands increase. LaGrone and Spieler (2006) also reported that older adults are more strongly influenced by name agreement. The present results extend their findings with a more refined stimulus set that distinguishes purely lexical selection from lexical and semantic selection. These results contrast with prior studies in which semantic relatedness with recently named pictures (e.g., blocked cyclic naming) was used to induce semantic selection difficulty and age group effects were not observed (Belke and Meyer, 2007; Gordon and Cheimariou, 2013; Mulatti et al., 2014), suggesting that naming multiple related pictures engages a more complex interplay of mechanisms and age differences.

## Experiment 3

As discussed in the Introduction, several studies have found that damage to the LIFG (or VLPFC) impairs selection in blocked cyclic naming (Schnur et al., 2006, 2009), verb generation (Thompson-Schill et al., 1998), and naming pictures with low name agreement (Kan and Thompson-Schill, 2004; Novick et al., 2009). Based on the robust selection demand (name agreement) effects demonstrated in Experiments 1 and 2 and the prior research on the role of LIFG in lexical-semantic selection, we predicted that participants with LIFG damage would have particular difficulty naming pictures in the Near Semantic Neighbors condition (relative to the High Name Agreement condition), and an analogous, perhaps somewhat smaller, effect for the Alternate Names condition.

### Methods

The experimental materials, procedure, and response coding were the same as in Experiments 1 and 2. The overall picture naming accuracy of participants with aphasia was somewhat lower than the neurologically intact participants in Experiments 1 and 2, and their performance varied widely. In combination with possible speech apraxia, lower accuracy and wide variability make naming latency a much less reliable measure for participants with aphasia. Thus, we used multilevel logistic regression to analyze picture naming accuracy. (Results of naming latency analyses were consistent with the accuracy results reported here.)

#### Participants

Fourteen older adults with aphasia secondary to a single left hemisphere stroke were recruited from the Moss Rehabilitation Research Institute Research Registry and completed this experiment for payment. All participants with aphasia were in the chronic stage (more than 6 months post onset), were native English speakers, passed a basic audiometric screening, and reported normal or corrected-to-normal vision. Participants were divided into two groups based on the location of their brain lesion. The Anterior group (*N* = 6) had damage that was mostly restricted to the frontal lobe and anterior temporal lobe (ATL), particularly affecting the LIFG. The Posterior group (*N* = 7) had damage restricted to the parietal lobe and posterior temporal lobe, sparing the LIFG. One participant was excluded from analysis due to a diffuse lesion that affected both anterior and posterior regions (this participant also had extremely low accuracy in the task, and so was excluded from *post hoc* analyses as well). Detailed demographic, neuropsychological, and neurological information about the 13 participants included in analyses is presented in **Table 4** (Mirman et al., 2010). The Anterior and Posterior groups were approximately matched (all *t* < 1.0, *p* > 0.35) on overall aphasia severity [Western Aphasia Battery Aphasia Quotient (Kertesz, 1982): Anterior *M* = 86.6, Posterior *M* = 82.9], semantic deficit [Camel and Cactus Test (Bozeat et al., 2000) accuracy: Anterior *M* = 78.7, Posterior *M* = 76.6], picture naming ability [Philadelphia Naming Test (Roach et al., 1996) accuracy: Anterior *M* = 83.7, Posterior *M* = 75.3], and overall lesion volume (Anterior *M* = 63.6 cc, Posterior *M* = 65.4 cc).

**Table 4:** Demographic, neuropsychological, and neurological characteristics of participants with aphasia (Experiment 3).

									LIFG % Damage				
Participant	Lesion group	Gender	Age	MPO	WAB AQ	CCT	PNT	Lesion volume (cc)	Pars orbitalis	Pars triangularis	Pars opercularis	Anterior ROI	Posterior ROI
MR0083	Anterior	M	54	175	95.1	91	92.6	51	0%	0%	12%	16%	1%
MR0253	Anterior	M	69	258	86.6	89	74.9	138	53%	60%	88%	24%	4%
MR0419	Anterior	F	46	149	91.5	77	91.4	52	16%	57%	90%	14%	0%
MR1857	Anterior	F	77	79	90.2	80	81.7	18	20%	39%	0%	6%	0%
MR2289	Anterior	F	75	59	73.2	58	76.6	62	0%	36%	81%	13%	0%
MR2350	Anterior	M	51	71	83.2	77	85.1	61	16%	54%	98%	14%	0%
MR1088	Posterior	F	52	112	88.3	66	76.6	89	0%	0%	0%	0%	24%
MR1743	Posterior	M	56	81	99.3	84	87.4	52	0%	0%	0%	0%	9%
MR2180	Posterior	M	71	63	41.4	72	25.1	67	0%	0%	0%	0%	22%
MR2221	Posterior	F	36	63	78.7	81	93.1	64	0%	0%	0%	0%	23%
MR2378	Posterior	F	57	48	96.0	80	79.4	91	0%	0%	0%	0%	18%
MR2464	Posterior	M	65	39	84.7	78	82.9	61	0%	0%	0%	0%	20%
MR2667	Posterior	F	64	12	91.7	75	82.3	34	0%	0%	0%	0%	10%

*MPO, Months Post Onset; WAB AQ, Western Aphasia Battery Aphasia Quotient; CCT, Camel and Cactus Test; PNT, Philadelphia Naming Test*.

### Results

**FIGURE 5:**
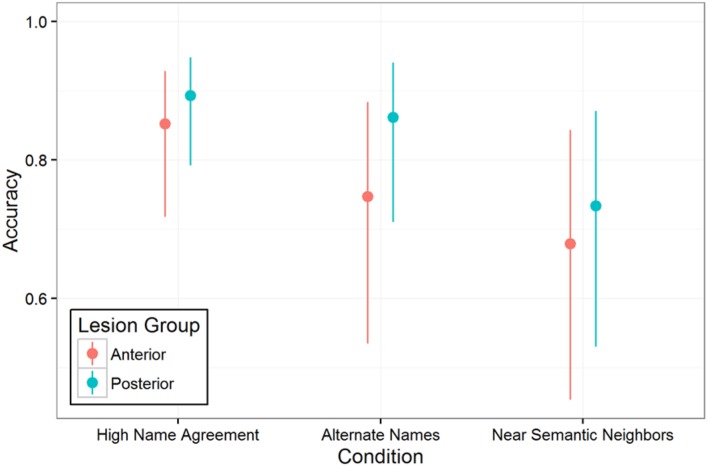
**Picture naming accuracy by condition and lesion group.** Vertical lines indicate 95% confidence intervals.

As shown in **Figure 5**, participants with aphasia had lowest accuracy when naming pictures in the Near Semantic Neighbors condition (High Name Agreement vs. Near Semantic Neighbors: *Estimate* = -1.1, *SE* = 0.41, *p* = 0.01; the other pairwise comparisons were not statistically significant, both *p* > 0.2). The main effect of lesion group was not statistically significant (*Estimate* = -0.22, *SE* = 0.22, *p* = 0.33), which was expected because the groups were approximately matched on aphasia severity and overall picture naming ability. Critically and surprisingly, the predicted interaction between lesion group and condition was also not statistically significant [χ^2^(2) = 1.09, *p* = 0.58]. That is, individuals with LIFG damage had no more difficulty naming pictures that required additional lexical selection (Alternate Names condition) or lexical and semantic selection (Near Semantic Neighbors condition) than did individuals with left hemisphere strokes that spared LIFG.

**FIGURE 6:**
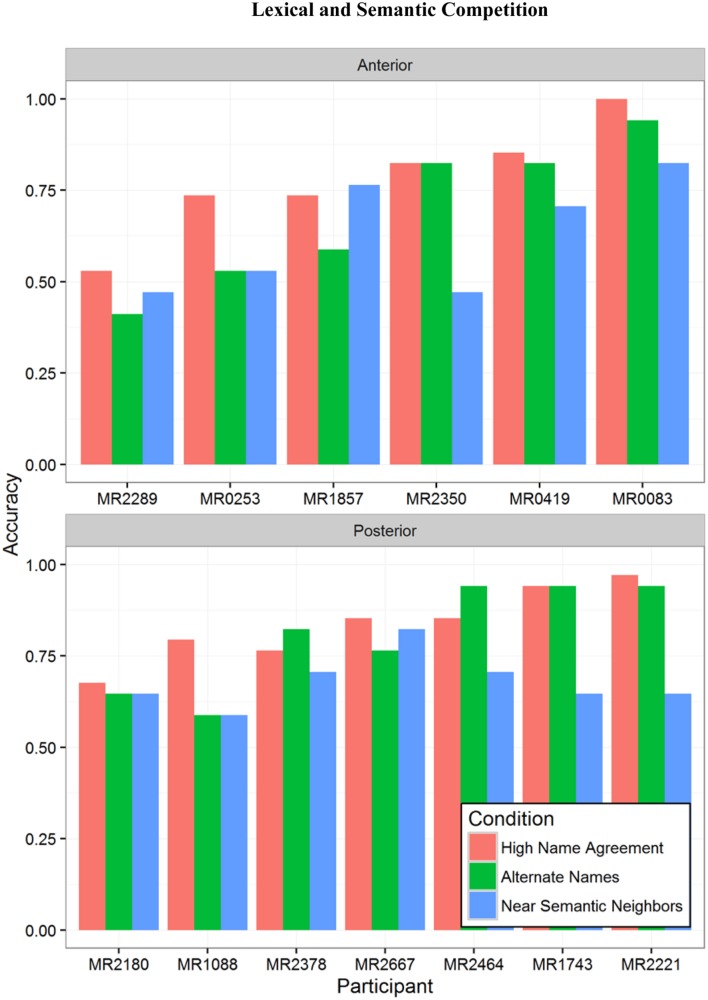
**Picture naming accuracy for individual participants with aphasia by condition.** Participants are ordered by overall accuracy. **(Top)** Shows Anterior group participants, **(Bottom)** shows Posterior group participants.

Picture naming performance by condition for individual participants with aphasia is shown in **Figure 6**, with the Anterior group in the top panel and Posterior group in the bottom panel. It is clear that some individuals exhibited substantially more sensitivity to the name agreement manipulation, but these individuals are about evenly split between the Anterior group and the Posterior group. Examination of **Figure 6** suggested that the individuals with better picture naming may be more sensitive to additional selection demands of naming low name agreement pictures, which goes against the typical observation that impairment severity has a more pronounced effect in more difficult conditions.

**FIGURE 7:**
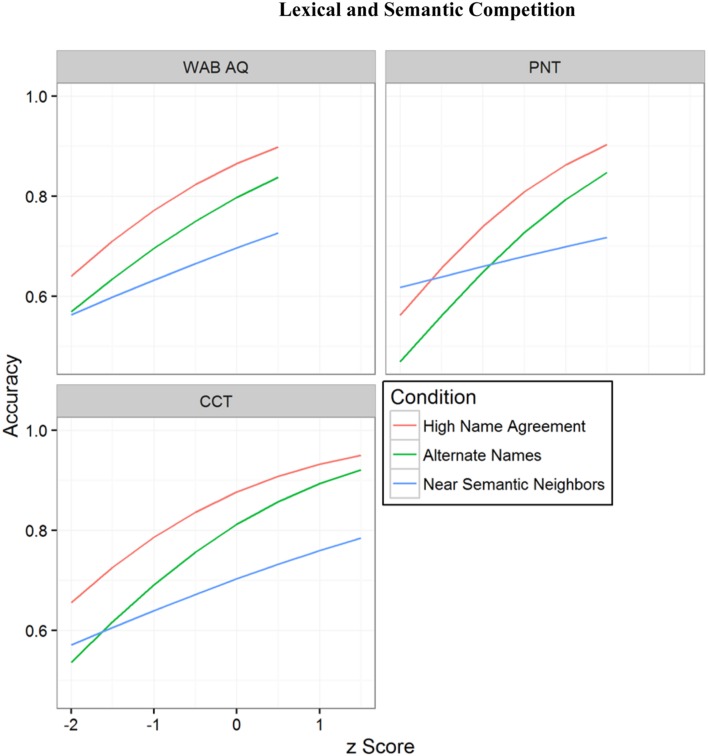
**Model-predicted picture naming accuracy for each condition as a function of each neuropsychological predictor**.

We conducted a series of exploratory *post hoc* analyses to evaluate whether neuropsychological factors such as aphasia severity (WAB AQ), semantic deficit (Camel and Cactus Test), or picture naming ability (PNT accuracy) predict differences in performance across condition. For these *post hoc* analyses, the key predictors (WAB AQ, PNT accuracy, and CCT accuracy) were z-transformed to create a standardized performance score and were tested individually as simple effects on naming High Name Agreement pictures and, critically, interactions with condition. That is, whether each of these predictors modulated the effect of condition on accuracy. Not surprisingly, each of the predictors was significantly associated with better naming performance in the High Name Agreement condition (WAB AQ: *Estimate* = 0.641, *SE* = 0.308, *p* = 0.038; PNT: *Estimate* = 0.796, *SE* = 0.328, *p* = 0.015; CCT: *Estimate* = 0.658, *SE* = 0.210, *p* = 0.002). None of these predictors was associated with differences between naming performance in the High Name Agreement condition vs. the Alternate Names condition (i.e., predictor-by-condition interaction; all *p* > 0.7). Differences between the High Name Agreement condition and the Near Semantic Neighbors condition were not significantly associated with WAB AQ (*p* > 0.1), were marginally associated with CCT performance (*Estimate* = -0.370, *SE* = 0.209, *p* = 0.077), and were statistically significantly associated with PNT performance (*Estimate* = -0.613, *SE* = 0.299, *p* = 0.040). The model-predicted relationships between performance on these predictors and picture naming accuracy for each condition are shown in **Figure 7**. A common pattern emerged: each predictor was associated with better performance for High Name Agreement and Alternate Names pictures, but less so for Near Semantic Neighbors pictures. This was particularly true for PNT and to a lesser degree for CCT and an ever lesser degree for WAB AQ. One interpretation of these results is that lexical-semantic selection demands are more relevant when semantic processing and naming ability are relatively intact enough to activate multiple candidates for selection; when they are impaired, candidates may not become sufficiently active for selection to be a substantive issue.

### Discussion

Based on prior research (Thompson-Schill et al., 1998; Schnur et al., 2006, 2009; Novick et al., 2009), we predicted that individuals with LIFG damage should have particular difficulty naming low name agreement pictures that require more difficult lexical and semantic selection. In a comparison of individuals with aphasia following left hemisphere strokes we found that, surprisingly, those with LIFG damage were no more sensitive to name agreement than those without LIFG damage. The groups were matched on aphasia severity, semantic deficit, naming ability, and lesion volume, so these other factors are unlikely to explain this null result.

It is possible that this experiment simply lacked the statistical power to detect an effect of LIFG damage, but there are several factors that cast doubt on this interpretation. First, the effect of name agreement was quite large – about 300 ms between each condition for the younger neurologically intact participants and even larger for the older neurologically intact participants. Second, there was a fairly strong effect of name agreement on accuracy for the participants with aphasia (81.0% correct for High Name Agreement pictures, 75.1% correct for Alternate Names pictures, and 65.6% correct for Near Semantic Neighbors pictures) and the naming performance was neither near ceiling nor near floor. These effects of name agreement on latency in neurologically intact participants and on accuracy in participants with aphasia were comparable to or larger than effects of other manipulations of lexical-semantic selection and competition that have been shown to be sensitive to LIFG damage. For example, after four cycles (the typical maximum), blocked cyclic naming effects reached about 80 ms in neurologically intact controls and the accuracy difference in participants with aphasia for heterogeneous vs. homogenous blocks was about 74% correct vs. 69% correct (Schnur et al., 2006) – effects that are half the size of the effects we report here. That is, in the present study, the effects of name agreement were large; they were just not modulated by LIFG damage.

Another legitimate concern is our small sample size – only six participants with LIFG damage and seven participants without. Although small, this sample of 13 participants is comparable with other studies that have shown effects of LIFG damage (e.g., *N* = 12 for anatomical analysis in Schnur et al., 2009; *N* = 7 left prefrontal lesion participants in Riès et al., 2014 and *N* = 6 in Riès et al., 2015; *N* = 14 total, *N* = 4 with LIFG damage, in Thompson-Schill et al., 1998). A related criticism is that not all of the participants in our Anterior group had very severe damage to LIFG and they varied in which part of LIFG was affected (e.g., MR1857 had no damage to pars opercularis, but pars opercularis was the only part of LIFG affected in MR0083). However, this does not explain why some of the largest effects of name agreement were exhibited by participants in the Posterior group (e.g., MR1088, MR1743, and MR2221), who had no damage to any part of LIFG. In short, although our failure to find an effect of LIFG damage could be partly due to lack of statistical power, the same criticism would apply even more strongly to studies that did find an effect of LIFG damage, so a better explanation is needed.

In a recent study of the role of prefrontal cortex (PFC) in selection, Riès et al. (2015) found that PFC damage was associated with larger blocked cyclic naming effects but not larger cumulative semantic interference effects. One possibility is that LIFG is particularly important when task structure allows (or even requires) “proactive” control or pre-selection (see also Belke and Stielow, 2013; Riès et al., 2014, 2015). For example, effects of LIFG damage on selection have been shown in blocked cyclic naming and verb generation: in blocked cyclic naming, there is a repeated set of items and selection must happen among those items; in verb generation, verbs can be pre-selected. In a recent eye-tracking study (Nozari et al., 2016), we found that LIFG damage impaired the rapid pre-selection that happens during sentence comprehension: using verb constraints to anticipate the final noun in sentences like “She will peel the banana.” In contrast, in the cumulative semantic interference paradigm and in the present underdetermined picture naming experiment, there is neither need nor opportunity to pre-select or proactively bias selection, which may explain why LIFG damage seems not to affect performance in these tasks.

If the importance of LIFG is related to pre-selection or anticipation processes, what neural systems might support the kind of selection required in our underdetermined naming task (and, presumably, cumulative semantic interference)? First, a substantial and growing set of studies indicates that left ATL damage has a particularly pronounced effect on semantically driven lexical access (Lambon Ralph et al., 2001; Mesulam et al., 2009; Schwartz et al., 2009; Walker et al., 2011; Mirman et al., 2015a,b). This aligns with our observation that, instead of LIFG damage, general picture naming accuracy was the best predictor of sensitivity to name agreement. That is, selection between equally valid names for a picture may be intrinsic to the semantically driven word retrieval system with limited engagement of additional (LIFG-mediated) selection systems. Second, white matter pathways are increasingly being recognized as playing a critical role in language and semantic processing (e.g., Papagno et al., 2011; Almairac et al., 2014; Duffau, 2015; Mirman et al., 2015a,b), though the mechanistic nature of that role (or roles) remains an open question.

## Conclusion

Word production requires rapid selection of specific words to convey the speaker’s message from 1000s (perhaps tens of thousands) of possible candidates. We investigated this selection process in the context of a picture naming task in which the critical pictures had multiple acceptable names. In a preceding norming study, those names were either judged to mean the same thing (Alternate Names condition) or to have distinct, closely related meanings that are appropriate for the picture (Near Semantic Neighbors condition). In Experiment 1, we found that young, neurologically intact adults were fastest to name pictures that only had one appropriate name (High Name Agreement condition), slower when selecting among synonymous names (Alternate Names condition), and slowest when selecting between closely related names (Near Semantic Neighbors condition). If there were a single selection stage, then naming latency should be a monotonic function of lexical-semantic similarity and Alternate Names pictures should be named more slowly than Near Semantic Neighbors pictures. The Experiment 1 results were the opposite, consistent with an alternative hypothesis that there are distinct selection processes at lexical and semantic levels. On this view, Alternate Names pictures require only lexical selection because the semantic representations are equivalent, but Near Semantic Neighbors require two stages of selection (semantic and lexical), thus producing slower naming latencies. There was also a strikingly higher rate of semantic and superordinate errors for Near Semantic Neighbors pictures further indicating increased difficulty at the semantic level.

Experiment 2 examined which, if any, of these selection stages are affected in typical aging by comparing naming latencies (and accuracy) of older adults (mean age: 65) against the younger adult pattern from Experiment 1. In addition to a main effect of age (slower picture naming for older adults compared to younger adults), there was an age group by condition interaction: age-related slowing was exaggerated in the Near Semantic Neighbors condition. The age group effect for the Alternate Names condition was intermediate: larger than for the High Name Agreement condition but smaller than for the Near Semantic Neighbors condition, and not statistically significantly different from either. These results suggest that age-related selection difficulties are more pronounced at the semantic level or perhaps build up across lexical and semantic levels.

Selection processes in language tasks have been associated with the LIFG (also called VLPFC). This association has been demonstrated in functional neuroimaging studies of neurologically intact adults (increased LIFG activation under high selection demands) and in neuropsychological studies (LIFG damage associated with greater sensitivity to selection demands). We predicted the same pattern for a comparison of individuals with left hemisphere stroke that either affected LIFG or spared it. We observed large effects of selection demand in the participants with left hemisphere stroke, but, surprisingly, whether or not the lesion included LIFG did not modulate the size of the selection demand effect. Although lack of statistical power is a possible concern, the selection effect itself was relatively large and our study sample size was comparable to other studies of effects of LIFG damage on selection, casting doubt on a simple power interpretation. An alternative interpretation is that LIFG is involved in proactive inhibitory control or pre-selection, which is not critical for the selection involved in underdetermined picture naming.

There is an ongoing and active debate about whether word production involves a lexical-semantic selection by competition (e.g., Levelt et al., 1999; Roelofs, 2003) or non-competitive lexical retrieval combined with a response monitor to exclude incorrect responses (e.g., Mahon et al., 2007). The present study provides new results that are consistent with selection by competition models: alternative responses compete and therefore produce slower responses. Many prior studies have used blocked cyclic naming or cumulative semantic interference paradigms and found similar evidence; however, those results can be explained in terms of competitive learning rather than competitive selection (Oppenheim et al., 2010). In the present studies, the selection demands were trial-specific and cannot be explained by competitive learning. Several studies have also used picture-word interference or other Stroop-like paradigms, in which selection (or monitoring) demands are directly increased by presenting a distractor word along with the target picture or color to be named. Some of these studies have found facilitative effects of related distractors, which some have interpreted in terms of a “horse-race” model in which selection is a matter of which representation crosses the response threshold first and no competition is required (e.g., Mahon et al., 2007). Such non-competitive selection models explain inhibitory (interference) effects as response monitoring – the interference arises at a later decision stage where distractors are excluded as incorrect responses. However, in the present study the co-activated alternatives were all correct responses and there was no need to exclude any of them. Response monitoring may play a role in the Near Semantic Neighbors condition (e.g., the monitor may be engaged to reject “tortoise” in favor of “turtle”), but it would not explain the inhibitory effect for the Alternate Names condition – if “sofa” happened to cross the threshold first, there is no reason for this response to be excluded in favor of “couch.” Indeed, on the non-competitive selection view, responses should have been faster in the Alternate Names condition than the High Name Agreement condition because it had multiple possible winners.

In sum, the present experiments show evidence of distinct selection processes at lexical and semantic levels that produce additive selection costs and differences in error type distributions. Compared to younger adults, older adults have particular difficulty when both lexical and semantic selection demands are increased. Finally, and surprisingly, LIFG damage was not associated with increased selection costs, suggesting that LIFG selection processes may be limited to proactive pre-selection processes.

## Author Contributions

The study was conceived by DM and designed in collaboration with AB and CF. Data were collected and coded by AB and CF, and analyzed by AB and DM. The manuscript was drafted by DM and AB with critical contributions from CF. All authors reviewed and revised the manuscript.

## Conflict of Interest Statement

The authors declare that the research was conducted in the absence of any commercial or financial relationships that could be construed as a potential conflict of interest.

## Footnotes

1 For example, 75% of participants labeled one item “present” (frequency = 89.45, length = 7); the remaining 25% used the label “gift” (frequency = 64.51, length = 4). The length and frequency of each response was multiplied by the percentage of participants who provided that response, and all weighted frequencies were added together to determine the final length and frequency for an item (e.g., present/gift frequency = 89.45^∗^0.75 + 64.51^∗^0.25 = 83.215).

2 This wide age range is similar to prior studies (LaGrone and Spieler (2006): *M* = 71.6, *SD* = 4.2; Mulatti et al. (2014) range 60–80) and Belke and Meyer (2007) found no differences between a “young-old” group (52–64) and an “old-old” group (68–88). Therefore, we did not find sufficient justification for a narrower restriction of the age range for older adults.

3 That the age group effect is smaller in the Near Semantic Neighbors condition than the High Name Agreement condition may not be immediately apparent in **Figure 4**, which shows accuracy plotted on the familiar linear proportion scale. Logistic regression operates on a logit scale, where differences near the bounds of the range are much larger. For an intuitive understanding, a difference of 1% vs. 2% errors is twice as many errors, but a difference of 10% vs. 11% errors is only a small proportional increase. For a more thorough discussion, see Jaeger (2008).
