# Supplementary material for: Distinct Effects of Lexical and Semantic Competition during Picture Naming in Younger Adults, Older Adults, and People with Aphasia
Source: Front Psychol. 2016 Jun 2;7:813. doi: 10.3389/fpsyg.2016.00813 (PMC4937813; doi:10.3389/fpsyg.2016.00813)
Supplement: Supplementary file 1 [file Table_1.DOCX]

Supplementary Material

Lexical and Semantic Competition in Word Production

Allison Britt1, Casey Ferrara2, Daniel Mirman1,2*

*** Correspondence:** Daniel Mirman, Department of Psychology, Drexel University, Philadelphia, PA, 19104, USA. dan@danmirman.org

# Supplementary Table: All Low Name Agreement Stimuli

| **Condition** | **Picture** | **Accepted Responses (Provided by More than 1 Participant in Name Agreement Norming Study)** |
| --- | --- | --- |
| Alternate Names | 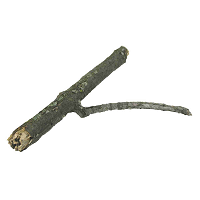 | Branch, stick, twig |
|  | 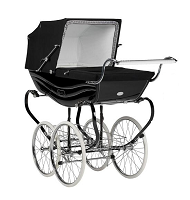 | Carriage, stroller, buggy, pram |
|  | 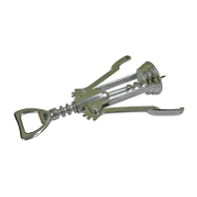 | Corkscrew, wine opener* |
|  | 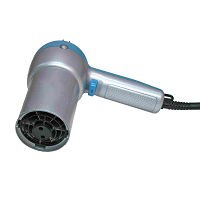 | Hair dryer, blow dryer, hair blower |
|  | 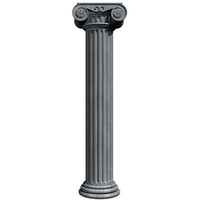 | Pillar, column |
|  | 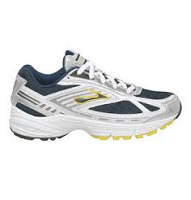 | Sneaker, shoe |
|  | 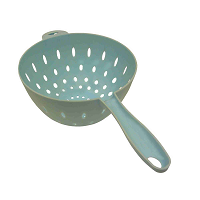 | Strainer, drainer, colander |
|  | 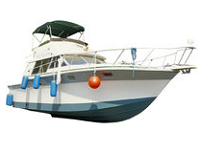 | Boat, yacht, ship* |
|  | 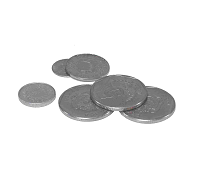 | Coins, change* |
|  | 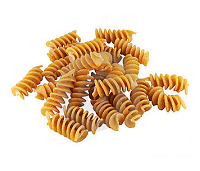 | Pasta, noodles, macaroni* |
|  | 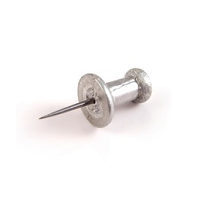 | Pushpin, thumbtack |
|  | 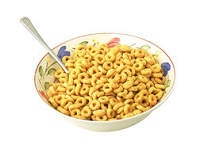 | Cereal, cheerios* |
|  | 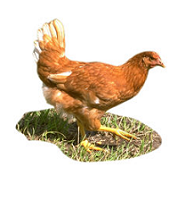 | Chicken, hen |
|  | 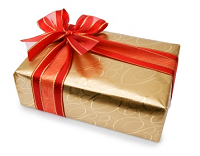 | Present, gift |
|  | 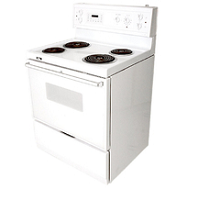 | Stove, oven |
|  | 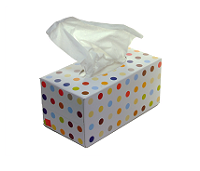 | Tissues, Kleenex* |
|  | 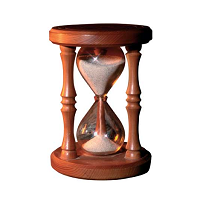 | Hourglass, timer* |
| Near Semantic Neighbors | 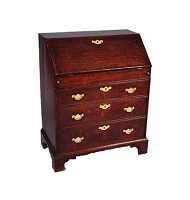 | Dresser, desk, cabinet, chest |
|  | 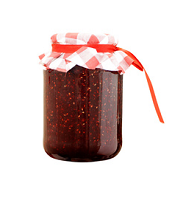 | Jam, jelly |
|  | 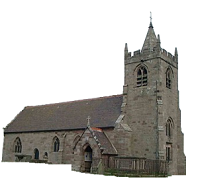 | Church, castle |
|  | 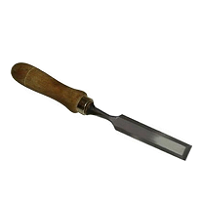 | Chisel, file* |
|  | 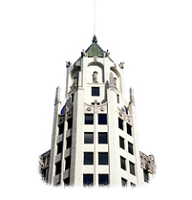 | Tower, church* |
|  | 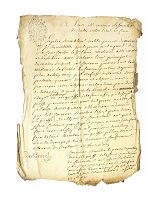 | Letter, document, paper, parchment |
|  | 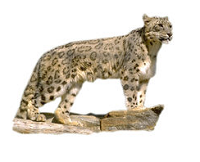 | Cheetah, leopard |
|  | 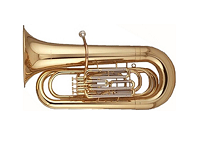 | Tuba, horn, trombone, trumpet |
|  | 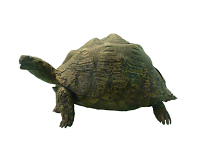 | Turtle, tortoise* |
|  | 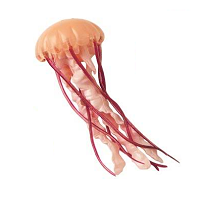 | Jellyfish, squid* |
|  | 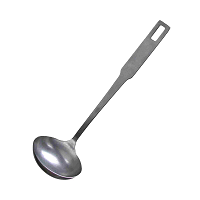 | Ladle, spoon |
|  | 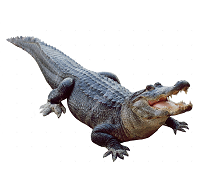 | Alligator, crocodile |
|  | 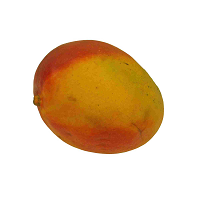 | Mango, peach* |
|  | 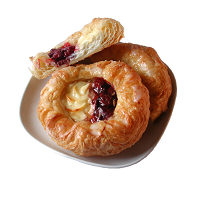 | Pastry, Danish, croissant |
|  | 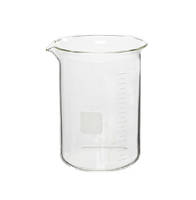 | Beaker, cup, measuring cup* |
|  | 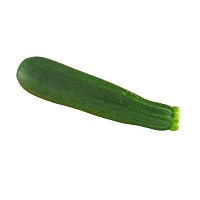 | Zucchini, cucumber |
|  | 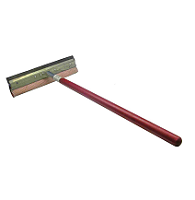 | Squeegee, mop* |

*Met condition criteria for previous norming study but not for results from current participants
